# Electronic cigarettes: “wolves in sheep's clothing”

**DOI:** 10.1016/j.jped.2024.06.015

**Published:** 2024-09-05

**Authors:** Débora Carla Chong-Silva, Maria de Fátima Bazhuni Pombo Sant'Anna, Carlos Antônio Riedi, Clémax Couto Sant'Anna, José Dirceu Ribeiro, Lais Meirelles Nicoliello Vieira, Leonardo Araújo Pinto, Regina Terse-Ramos, Mariana Aparecida Pasa Morgan, Ricardo Neves Godinho, Renata Cantisani di Francesco, Carlos Augusto Mello da Silva, Marilyn Urrutia-Pereira, João Paulo Becker Lotufo, Luciana Rodrigues Silva, Dirceu Solé

**Affiliations:** a Sociedade Brasileira de Pediatria (SBP), Departamento Científico de Pneumologia, Curitiba, PR, Brazil; b Sociedade Brasileira de Pediatria (SBP), Departamento Científico de Pneumologia, Rio de Janeiro, RJ, Brazil; c Sociedade Brasileira de Pediatria (SBP), Departamento Científico de Pneumologia, Campinas, SP, Brazil; d Sociedade Brasileira de Pediatria (SBP), Departamento Científico de Pneumologia, Belo Horizonte, MG, Brazil; e Sociedade Brasileira de Pediatria (SBP), Departamento Científico de Pneumologia, Porto Alegre, RS, Brazil; f Sociedade Brasileira de Pediatria (SBP), Departamento Científico de Pneumologia, Salvador, BA, Brazil; g Universidade Federal do Paraná, Programa de Pós-Graduação em Saúde da Criança e do Adolescente, Curitiba, PR, Brazil; h Sociedade Brasileira de Pediatria (SBP), Departamento Científico de Otorrinolaringologia, Belo Horizonte, MG, Brazil; i Sociedade Brasileira de Pediatria (SBP), Departamento Científico de Otorrinolaringologia, São Paulo, SP, Brazil; j Sociedade Brasileira de Pediatria (SBP), Certificado em Toxicologia Médica – Departamento Científico de Toxicologia e Saúde Ambiental, Porto Alegre, RS, Brazil; k Sociedade Brasileira de Pediatria (SBP), Departamento Científico de Toxicologia e Saúde Ambiental, Uruguaiana, RS, Brazil; l Sociedade Brasileira de Pediatria (SBP), Grupo de Trabalho sobre Drogas e Violência em Adolescentes, São Paulo, SP, Brazil; m Vice-Diretor Científico - Sociedade Brasileira de Pediatria (SBP), Salvador, BA, Brazil; n Diretor Científico - Sociedade Brasileira de Pediatria (SBP), São Paulo, SP, Brazil

**Keywords:** Electronic cigarettes, Electronic nicotine delivery systems, Adolescent, Risks, Acute lung injury

## Abstract

**Objective:**

To provide cutting-edge information on the impact and risks of using Electronic Nicotine Delivery Systems (ENDS) by children and adolescents, based on the latest evidence published in the literature.

**Data source:**

A comprehensive search was carried out on PubMed, using the expressions ‘‘electronic cigarettes’’ OR ‘‘electronic nicotine delivery systems” OR “vaping” AND ‘‘adolescent’’ AND “risks” AND ‘‘acute lung injury’. All retrieved articles had their titles and abstracts read to identify and fully read the papers reporting the most recent evidence on each subject.

**Summary of findings:**

The use of ENDS has alarmingly increased in Brazil and around the world. The possibility of customizing use, the choice of flavors and nicotine content, and the general notion that these devices are harmless when compared to conventional cigarettes are some of the factors responsible for this increase. Numerous scientific studies have proven that electronic cigarettes have serious consequences for the respiratory system, such as EVALI (E-cigarette or Vaping-Associated Lung Injury) and difficult-to-control asthma, as well as harmful effects on the neurological, cardiovascular, gastrointestinal, and immunological systems. High concentrations of nicotine make many young people addicted to this substance. In Brazil, commercialization, import, and advertising are prohibited. The viable interventions to address the use of these devices in children and adolescents are prevention and behavioral counseling.

**Conclusion:**

There is clear scientific evidence that these devices pose a risk to the physical and mental health of children and adolescents.

## Introduction

Since its appearance on the American market in 2007,^1^ the use of electronic cigarettes, also known as ‘e-cigs’, ‘vapes’, and ‘pods’, has rapidly grown in popularity among young people and adults worldwide, including in Brazil.^2^^,^^3^

Scientifically named Electronic Nicotine Delivery Systems (ENDS), their use has been driven by the possibility of personalizing use, choosing flavors and nicotine content, and the general notion that e-cigs are harmless compared to conventional cigarettes, which is the mistaken theory of “harm reduction”.^4^

Proponents of their use still support the hypothesis that e-cigs are effectivesmoking cessation tools.^4^ However, available research shows just the opposite, indicating that young people who start smoking with electronic cigarettes are more likely to switch to conventional cigarettes compared to those who have never used these devices.^5^ In these cases, the chance of starting to use conventional cigarettes is approximately three times greater.^6^

Another important factor is the dual use of conventional cigarettes and e-cigs. A study with young Europeans aged 15 to 16 years demonstrated a 6.4% rate of dual-use (regular and electronic cigarettes),^7^ and an even higher number in six Nordic countries of 23 to 31%.^8^

The 2019 National Health Survey (PNS) shows that around 70% of ENDS users are teenagers and young adults aged between 15 and 24 years old, including those who have never smoked common cigarettes. The National School Health Survey (PeNSE), which gathers data collected between 2009 and 2019, shows that 16.8% of students between 13 and 17 years old have tried electronic devices at least once, with a trend of greater consumption among boys.^2^^,^^9^

According to a report from the World Health Organization (WHO) from December 2023, the number of young people aged 16 to 19 using electronic cigarettes doubled from 2017 to 2022 in Canada and tripled in England in the last three years. Furthermore, data from the Global Tobacco Surveillance System reveals that the use of ENDS among young people aged 13 to 15 exceeds that of those over 15 years old, and in countries like Paraguay, this number is four times higher.^10^

## Sources

A narrative literature review on the subject was carried out using different types of documents, especially scientific articles, but also documents from regulatory agencies and legislative projects, as well as print and online media reports.

The objective of this method was to provide a broad and updated description of the subject, without necessarily exhausting all sources of information, as a systematic search and analysis of data was not conducted. Its importance lies in the availability of current data on the topic, given the urgency and relevance of the subject.

In the literature search, the following descriptors were used: “Electronic cigarettes,” “Electronic Nicotine Delivery Systems,” “Adolescent,” “Risks,” “Acute Lung Injury” in the PubMed electronic databases.

A specific search was carried out in the main world regulatory agencies: National Health Surveillance Agency (ANVISA, https://www.gov.br/anvisa/pt-br), U.S. Food and Drug Administration (FDA, https://www.fda.gov), European Medicines Agency (EMA, https://www.ema.europa.eu/en/homepage), as well as on the websites of Scientific Societies in Brazil and worldwide – American Thoracic Society (ATS, https://site.thoracic.org), European Respiratory Society (ERS, https://www.ersnet.org), American Academy of Pediatrics (AAP, https://www.aap.org), Pan American Health Organization (PAHO, https://www.paho.org/pt/brasil), World Health Organization Framework Convention on Tobacco Control (WHO FCTC, https://fctc.who.int/who-fctc/overview), Brazilian Society of Pulmonology and Phthisiology (https://sbpt.org.br/portal/), Brazilian Society of Pediatrics (https://www.sbp.com.br), National Cancer Institute (INCA, https://www.gov.br/inca/pt-br), Oswaldo Cruz Foundation (FIOCRUZ, https://portal.fiocruz.br), and Brazilian Medical Association (AMB, https://amb.org.br).

Scientific studies that presented the described method in a detailed and comprehensive way and clear results were selected to contribute to the review.

## Summary of findings

### *What are electronic nicotine delivery systems?*

ENDS, in their most basic form, are devices consisting of a circuit with a reservoir containing a liquid solution, an atomizer to generate heat, an energy source (usually a battery), and a nozzle for consumption. When activated, the device generates heat, and the fluid passing through the atomizer turns into an aerosol or vapor that can be inhaled by the user.^4^

Over the years, these devices have been “perfected” to become more attractive and functional. In the first generation, electronic cigarettes were designed to mimic the appearance of regular cigarettes and were known as *‘cigalikes’.* They typically consisted of a battery, an atomizer, and a reservoir. They were designed to be discarded after the end of fluids, whose nicotine concentrations were variable and had different aromas.^5^ The second generation of devices entered the market with the same basic circuit but featured a longer-lasting, rechargeable battery and a replaceable fluid reservoir. Additionally, the *design* evolved to take different forms, moving away from the conventional cigarette look. The improved electrical circuit enhanced the modulation of the puff and consequently the delivery of nicotine.^5^^,^^11^ The third generation of ENDS introduced a low-resistance atomizer that generates greater heat, improving the quality of the aerosol and producing a stronger release or dose of nicotine and other substances.^11^

The fourth generation comprises the most current devices, called ‘*pod*’ or ‘*pod-mods*’. These use a cartridge or capsule with liquid that contains the same amount of nicotine as 20 or more cigarettes and can be refillable or disposable. They typically use nicotine salts, which have a lower pH than free-base nicotine, thereby increasing inhaled nicotine levels and causing less throat irritation. Because they are small, resembling a *USB flash drive* (although they can take on various shapes, such as a comma or diamond) they are easily transported and hidden, making them one of the most popular devices among teenagers.^12^^,^^13^ Vaporizers, also part of this generation, are devices used to inhale active substances such as dried herbs, wax, and oil.^12^

Due to the rapidity with which new products are released, newer devices may not fit neatly into the “fourth generation” category, just as products from previous generations may fall into more than one category. Regardless, the market remains fast-paced, with countless options and new features for consumers, and the literature is unable to study them with the same agility.^6^^,^^13^

The wide variety of models and flavorings and the possibility of discreet use make these devices attractive and popular among young people, who often do not realize that the cartridges contain nicotine.^4^ The aroma released by the device also makes the idea of smoking socially acceptable and pleasant, as well as arousing the curiosity of those around.^4^^,^^13^

In 2014, a different generation of ENDS was launched on the Japanese market, the so-called *Quit-Ordinary-Smoking* (iQOS®), which falls into the class of ‘heated tobacco products’ (HTPs). These devices heat tobacco instead of burning it, to supposedly deliver an aerosol with fewer toxic chemicals than cigarette smoke. iQOS® consists of three main components: a disposable tobacco stick (called *a HeatStick*), a battery-powered heating pad, and a charger. The tobacco stick is heated to a maximum temperature of 350 °C (a common cigarette is burned above 600 °C) for around six minutes, providing 12 to 14 puffs.^14^^,^^15^

### *The beginning*

The creation of the electronic cigarette is frequently credited in informal literature to the Chinese pharmacist Hon Lik and dates back to 2003. Lik, who graduated from the Liaoning Traditional Chinese Medicine College, sought the “disruptive technology” that could compete with conventional cigarettes, as he was a heavy smoker and had already lost his father to lung cancer.^16^, ^17^, ^18^

The idea reportedly came to Lik when he woke up after a night full of nightmares, despite using a nicotine patch while sleeping. This made him reflect that the only satisfactory way to provide nicotine in an adequate and effective form that could make users abandon conventional cigarettes would be through some form of smoking.^17^

Ten years later, in 2013, Fontem Ventures, the Dutch subsidiary of Imperial Tobacco of the United Kingdom, bought the patents of Dragonite International Limited, from Hong Kong, for US$75 million. This company was co-founded by Hon Lik together with a Chinese investor.^16^^,^^17^

Although Hon Lik is known as the “father” of the electronic cigarette, records show that many years earlier, as early as 1963, cigarette companies were working on devices that did not burn tobacco, in an effort to develop cigarette alternatives with “reduced harm” or that were “socially acceptable”. These efforts included products that heat tobacco instead of burning it, such as the Ariel cigarette from the 1960s by British American Tobacco, Premier from the 1980s, and Eclipse from the 1990s, both by RJ Reynolds, and Accord from the 1990s/2000s by Philip Morris. None of these products achieved commercial success, and their similarities to current electronic cigarettes were minimal, as they did not heat a liquid nicotine solution.^16^

Finally, in 2013, NuMark, a subsidiary of Philip Morris, launched the MarkTen electronic cigarette based on nicotine aerosol technology that had been developing since 1990.^16^

### *The risks of using ENDS / electronic cigarettes*

The risks of using ENDS are numerous and begin in the circuit that houses the fluid. Containers can have different compositions such as nickel-chromium, chromium-aluminum-iron alloys, and other metals such as copper, silver, zinc, and tin. The constant heating and cooling of these metallic components can leave traces of particles in the fluid that will be inhaled. The high temperature to which the atomizer is subjected can produce volatile carbonyl species that contribute to toxicity.^19^ Previous studies with welders have proven the systemic toxicity of inhaling metal vapors. The recorded consequences include an increased frequency of respiratory tract infections, immune suppression, lung damage, and an increased risk of lung cancer.^20^

The lithium batteries that power the system are also susceptible to explosions and fires, especially during charging, which can cause burns or other thermal injuries.^20^

The fluid that completes the device is composed of varying proportions of vegetable glycerin, propylene glycol, nicotine, and flavoring agents. Both propylene glycol and glycerin can form toxic aldehydes when heated.^20^ Acetaldehyde, acrolein, and formaldehyde are the primary toxic aldehydes present, contributing to the risk of cardiovascular and pulmonary diseases. Research on different types of fluids has determined emissions of these aldehydes and other carbonyls from e-cigarette use, and they were found in the aerosols of all e-cigarettes. Additionally, newer-generation devices produce more aldehydes than first-generation devices due to higher battery power output.^21^ The nicotine concentration is variable and can exceed 59 mg/ml, although there is no guarantee of quality or the manufacturing process. For comparison purposes, a common cigarette provides around 2 mg of nicotine to the user. Therefore, 1 ml of vaporized fluid consumed can exceed the nicotine equivalent of a pack of conventional cigarettes.^20^

In the case of iQOS®, of marketing claims are eye-catching, with promises about the real taste of tobacco, no fire, no ash, less smoke, and reduced levels of toxic chemicals. The manufacturer claims that the product is less harmful than traditional cigarettes, but this assertion is supported by its own clinical data.^14^^,^^15^ Reported results show slight improvements for iQOS®, with 4 out of 13 biomarkers differing from those of conventional cigarettes (where one might expect one false positive by chance). However, these findings are not robust enough to justify concluding a reduced risk.^22^ In reality, iQOS® smoke contains carcinogenic by-products of pyrolysis and thermogenic degradation typical of traditional cigarettes, which can cause severe lung damage, increased risk of lung cancer, and harmful effects on the cardiovascular system. There is currently no evidence to show that HTPs are any less harmful than conventional cigarettes; rather, any nicotine product is addictive and carcinogenic.^14^^,^^15^^,^^23^

## Local damage from electronic cigarettes

**Central Nervous System (CNS)**

Studies showing neurotoxic effects, which directly affect neurodevelopment and cognition, as well as those leading to addiction, are robust and scientifically respected, despite mostly being animal studies. It becomes easy to understand these effects by knowing the components of e-liquids, such as solvents (over 75%) - usually propylene glycol and/or vegetable glycerin, water (20%), chemical flavorings (10%), nicotine (2%) and similar substances. Additionally, substances such as formaldehyde, acrolein, and numerous trace substances like heavy metals, phenolic compounds, and polycyclic aromatic hydrocarbons have been found. In light of this, it was found that the effects of e-cigs on brain functional connectivity at rest and withdrawal symptoms were similar to those observed with conventional cigarettes.^24^

### *Nicotine and addiction*

Nicotine is a highly addictive compound present in tobacco leaves. Inhaling the combustion products of tobacco is a rapid way to absorb nicotine into the lungs and make it available in the bloodstream.^25^ In the adolescent brain, nicotine affects the development of the reward system, as well as the circuits that control attention and learning, which can lead to the development of mood disorders and permanent problems with impulse control.^4^

Nicotine is capable of disturbing the balance of cholinergic transmission, resulting in behavioral impairments. The overall toxicity of nicotine related to cholinergic overstimulation is well-known, and many of nicotine's psychopharmacological effects are related to its addictive properties, with this substance provided by e-cigarettes being as or more addictive than that of conventional cigarettes.^26^ Nicotine stimulates the central reward pathway, known as the “mesolimbic reward pathway”.^24^ The importance of this pathway in conferring “pleasure” or “reward” through natural stimuli such as food or sex, or various addictive drugs, has been established for decades.^27^ Currently, 12 different subunits of neuronal nicotinic receptors are described (α2-α10 and β2-β4), with α4β2 subunits primarily involved in mediating nicotine's rewarding effects, as well as in addiction.^24^

Some recent studies have suggested that nicotine might have a protective effect against certain neurological diseases such as depression.^28^ However, these studies are not yet sufficient to support the idea that nicotine, a substance known to be neurotoxic and highly addictive, should be regularly used to treat these diseases.^24^

Due to the unknown origin of many products, labels may not accurately reflect the actual contents, obscuring the true nicotine content provided to the user. Some ENDS marketed as nicotine-free may have higher concentrations than those marketed as containing nicotine.^1^

### *The nicotine salt controversy*

In October 2018, after the *Food and Drug Administration* (FDA) took action against a rising electronic cigarette manufacturer, a major controversy ensued. Documents were seized, suggesting that the company engaged in practices aimed at enticing young people to consume tobacco products. Unlike other electronic cigarette brands that use freebase nicotine, this company utilized nicotine treated with benzoic acid, resulting in nicotine salts that can deliver concentrations of this compound up to 10 times higher to the user. While the tobacco industry has a history of conduct capable of increasing nicotine levels in cigarettes, the use of these salts in ENDS was unprecedented.^29^

Nicotine salt, formed by combining nicotine with benzoic acid, lowers the pH of e-liquid, creating smoother vapor and improving nicotine absorption in the lungs over extended periods. Nicotine salts expedite nicotine's journey to the brain, enhancing its impact on receptors and immediately inducing feelings of pleasure and well-being through dopamine release. However, prolonged exposure to benzoic acid can cause symptoms such as coughing, sore throat, abdominal pain, nausea, and vomiting.^30^^,^^31^

Despite regulatory efforts by the FDA, the introduction of salt nicotine into the e-cigarette market has significantly increased its popularity among youth and young adults. A systematic review of 48 articles, primarily from North America and Europe, examining regulations on electronic nicotine products, indicates that flavor restrictions notably decreased youth electronic nicotine product use, while taxation reduced adult use. Mixed results were found regarding the impacts of age restrictions. Overall, ongoing efforts and possibly stricter regulations are warranted to mitigate the allure and potential health risks associated with nicotine product use.^31^^,^^32^

### Cardiovascular System

Elevated levels of systemic oxidative stress pose a risk for the development of cardiovascular disease. There is evidence that this cytotoxicity is caused by flavorings and other additives present in e-cigarette fluid.^33^ The use of e-cigarettes is associated with an increase in resting heart rate, which is an independent risk factor for heart failure. Additionally, nicotine has a significant and potentially deleterious effect on other short-term cardiovascular hemodynamic parameters and biomarkers, leading to hypertension and increasing the risk of acute myocardial infarction and stroke. Studies also reveal an increased risk of thrombotic events due to platelet aggregation.^13^^,^^34^

Immediate physiological effects of e-cigarette use have been demonstrated, especially on the cardiovascular system, in both animal and human studies. The use of nicotine-containing e-cigarettes has been significantly associated with increased heart rate, systolic and diastolic blood pressure, and an increased Augmentation Index (AIx75), very similar to those observed after the use of conventional cigarettes. This suggests nicotine as the main culprit behind these effects.^35^

### Gastrointestinal System

The most common acute gastrointestinal symptoms associated with electronic cigarette use include epigastric pain, nausea, vomiting, diarrhea and hemorrhage, primarily caused by high levels of metals such as copper and chromium. In more chronic cases, there are reports of relapsing ulcerative colitis.^6^^,^^36^

### Respiratory System

Inhalation of particles can increase the permeability of the respiratory tract epithelium, leading to an influx of inflammatory cells, which can cause lung disorders such as bronchiolitis obliterans, pneumonitis, and acute eosinophilic pneumonia.^13^ The perpetuation of this process contributes to chronic inflammation and secretion of mediators that cause the destruction of lung tissue. There is also an increase in cell death due to oxidative stress even in the absence of nicotine.^33^

The immediate effect of e-cigarette use on the respiratory system has been measured, showing a significant decrease in the exhaled fraction of nitric oxide, indicating an early inflammatory effect, even after occasional use of the devices. Regarding the respiratory system, changes in spirometry were not immediately observed with use, suggesting that functional alterations should be evaluated in the medium and long term in regular users of the devices.^35^

The most recent diagnosis related to the use of electronic cigarettes involves alveolar damage, the so-called *E-cigarette or Vaping-Associated Lung Injury –* EVALI, discussed below*.*^37^

### Oral cavity

Nicotine in the oral cavity has been shown to alter the cytoskeleton and induce remodeling of the extracellular matrix of fibroblasts from the gums of common cigarette smokers. When exposed to vapor from electronic cigarette fluids, even those that do not contain nicotine, the production of reactive oxygen species still occurs, showing that it is not only nicotine that leads to changes in morphology, proliferation, apoptosis, and cell migration in the oral mucosa.^9^ Constant inflammation and damage can lead to oral ulcerations and cell mutations with an increased risk of oral cancer.^13^

Accidental or intentional ingestion of the fluid can cause serious adverse effects, which can be fatal depending on the formulation and concentration of nicotine present.^37^ Furthermore, chronic dermal exposure to fluid can cause contact dermatitis in the perioral area and eye irritation, mainly due to the presence of nickel and propylene glycol in the fluids.^36^

In the immune system, e-cigarette consumption is linked to the nonspecific activation of the inflammation cascade, reducing the effectiveness of the defense system and leaving patients more exposed to viral infections, including COVID-19, and pneumonia.^38^

Although toxic and carcinogenic metabolites are found in e-cigarette users, the long-term effects are still being studied. However, there is evidence suggesting that use may increase the risk of cancer. Chemicals present in aerosols, such as formaldehyde, acrolein, nickel, lead, and cadmium, are capable of causing DNA damage and mutagenesis, with potential carcinogenic effects.^39^ Table 1 contains a summary of the impact of e-cigs on the different systems.

**Table 1:** The impact of electronic cigarettes on organs and systems.^6^^,^^9^^,^^13^^,^^25^^,^^33^, ^34^, ^35^, ^36^, ^37^, ^38^, ^39^, ^40^^,^^42^, ^43^, ^44^

**ACTION OF ELECTRONIC CIGARETTES ON ORGANS AND SYSTEMS**		
**Organ**	**Action**	**Symptoms**
	• Cholinergic overstimulation • Neurotoxicity	• Addiction • Changing behavior • Cognitive and neurodevelopmental impairments • Stroke
	• Elevated oxidative stress • Nicotine has deleterious hemodynamic effects • Increased platelet aggregation	• Cardiovascular diseases • Increased resting heart rate • Increased blood pressure • Cardiac insufficiency • Hypertension • Acute myocardial infarction • Increase in thrombotic events
	• High levels of metals such as chromium and copper.	• Pain in epigastrium • Nausea • Vomiting • Diarrhea • Bleeding • Ulcerative colitis
	• Increased permeability of the respiratory tract epithelium. • Influx of inflammatory cells. • Drop in the fraction of exhaled FeNO.	• Early inflammatory process • Bronchiolitis obliterans • Pneumonitis • Acute eosinophilic pneumonia • Chronic inflammation • Destruction of lung tissue • Alveolar damage
	• Alteration of the cytoskeleton • Induction of extracellular matrix remodeling • Reactive oxygen species alter morphology, proliferation, apoptosis and cell migration in the oral mucosa	• Oral ulcers • Oral cancer
	• Nonspecific activation of the inflammation cascade, reducing the effectiveness of the immune system	• Greater exposure to viral infections

## Main associated respiratory diseases

### *E-cigarette or Vaping-Associated Lung* Injury - EVALI

Between 2019 and 2020, approximately 2800 cases of serious lung injury associated with the use of electronic cigarettes were reported in the United States of America (USA). This disease was termed *E-cigarette or Vaping-Associated Lung Injury* (EVALI) and had a significant impact on the perception of these devices. Subsequent investigations revealed it that the injuries were associated with the presence of tetrahydrocannabinol (THC) in conjunction with vitamin E acetate.^37^^,^^38^

A case series describing 53 patients with confirmed or probable EVALI in Wisconsin and Illinois provided a functional definition of respiratory failure with symptom onset within 90 days after e-cigarette use. Patients showed pulmonary infiltrates on imaging studies, absence of infection, and other causes of respiratory failure.^40^

EVALI is characterized by an acute pulmonary inflammatory process, resulting in alveolar collapse and severe impairment of gas exchange secondary to damage to the alveolar-capillary membrane. Its diagnosis is exclusionary, based on clinical history (e-cigarette use) and symptoms such as fever, cough, dyspnea, hemoptysis, headache, fatigue, nausea, and vomiting,^38^^,^^41^ with up to a third requiring intubation and mechanical ventilation.^42^

This can be corroborated by radiological findings that include diffuse bilateral infiltrates with ground-glass opacities, predominantly basal and subpleural sparing.^42^ Additionally, diffuse tree-in-bud and bilateral nodular infiltrates mimicking metastatic malignancy have also been described.^40^^,^^42^ A more recent review of imaging patterns suggests four radiographic patterns of EVALI, including acute eosinophilic pneumonia, diffuse alveolar damage, organizing pneumonia, and lipoid pneumonia.^43^

From a histological perspective, a recent review of lung biopsies from 17 EVALI patients showed patterns of acute lung injury in all cases. These patterns included acute fibrinous pneumonitis, diffuse alveolar damage, and organizing pneumonia.^44^ A summary of findings supporting the diagnosis can be seen in Table 2.

**Table 2:** Criteria suggestive of the diagnosis of EVALI. Adapted from Winnicka L & Shenoy MA. EVALI and the Pulmonary Toxicity of Electronic Cigarettes: A Review. J Gen Intern Med. 2020;35(7):2130–2135.^40^

**Suggested Diagnostic Criteria of EVALI**
Suggestive symptoms (fever, cough, dyspnea, hemoptysis, GI symptoms)
Vaping history in past 90 days
Suggestive laboratory work (leukocytosis, transaminitis, elevated ESR/CRP)
Exclusion of infection - *everyone*
Suggestive imaging (bilateral inflitrates, diffuse, ground-glass with basilar predominance and subpleural preservation. Diffuse tree-in-bud and bilateral nodular infiltrates have been described.)
Exclusion of cardiac, rheumatologic, and oncologic causes
Bronchoscopy with BAL (individual decision for each case)
Lung biopsy (individual decision for each case)

Reported cases of EVALI declined with the onset of the COVID-19 pandemic. However, confinement has led to life changes, such as an increase in smoking among teenagers and young adults. While EVALI may have become less prevalent due to increased public awareness and the removal of vitamin E acetate from *vaping products, recent* reports of EVALI cases following vaccination against SARS-CoV-2 serve as a reminder to healthcare professionals that the syndrome should remain in the differential diagnosis of young patients admitted with lung injury. During the pandemic, COVID-19 infection has been observed to be five times more likely in e-cigarette users and seven times more likely in users of both e-cigarette and conventional cigarettes. This increased susceptibility may attributed to diffuse alveolar damage and decreased secretory IgA in the nasal mucosa caused by EVALI, making individuals more vulnerable to COVID-19 infection.^38^ Additionally, the coronavirus primarily enters host cells through the angiotensin-converting enzyme 2 (ACE2) receptor, which is abundant on lung epithelial cells. Interestingly, smokers, including e-cigarette users, exhibit elevated ACE2 expression, potentially increasing their susceptibility to COVID-19. Furthermore, men generally have higher ACE2 levels, suggesting gender-based variations in disease severity.^45^

Similar to EVALI, COVID-19 patients have exhibited elevated levels of several cytokines in bronchoalveolar lavage fluid and peripheral blood mononuclear cells. The primary complication of SARS-CoV-2 infection, Acute Respiratory Distress Syndrome (ARDS), stems from a ‘cytokine storm’ triggered by the uncontrolled release of proinflammatory cytokines and chemokines from immune cells, including IL-6, TNF-α, IFN-γ, and others. Studies indicate that SARS-CoV-2 infection in bronchial epithelial cells suppresses IFN-mediated responses while augmenting cytokine and chemokine production, facilitating viral replication. Chronic smoking exacerbates this inflammatory response by reducing levels of perforin and granzyme B, critical proteins for NK and CD8 T cell function. Autopsy findings in COVID-19 patients have revealed neutrophil infiltration in lung capillaries, the formation of Neutrophil Extracellular Traps, and subsequent organ damage, suggesting that smoking may aggravate ARDS development and increase disease severity.^45^^,^^46^

It should also be noted that many individuals infected with COVID-19 do not exhibit any symptoms of the disease. Therefore, there is a possibility that vapers with EVALI could serve as asymptomatic carriers of COVID-19, potentially contributing to the spread of the disease. Enhanced recognition of at-risk individuals coupled with efforts aimed at ACE2 receptors (and their variations) and other immune and inflammatory targets could influence pandemic management and curb further infections.^45^

It is currently known that several substances may be associated with lung damage present in EVALI. The main ones are THC, nicotine, simultaneous use of THC and nicotine, vitamin E acetate, and cannabidiol (either independently or associated with one or more of the previous substances).^38^^,^^41^ The exact numbers of the disease are still uncertain, but it is established that the majority are male (67%), with an average age of 24 years, and up to 86% are associated with vaping products containing THC. Reporting exclusive use of THC and/or frequent use of THC (more than 5 times per day) and obtaining products from "informal" sources such as the street, a dealer, or a friend were the main reported related factors.^47^

Given the nearly infinite combinations of ENDS and fluids, pinpointing the exact causes of EVALI has been challenging.^38^^,^^41^

### Asthma

Asthma is a chronic inflammatory disease characterized by limitations in the passage of air through the bronchi, accompanied by other respiratory symptoms. It arises from complex interplay of genetic and environmental factors, including pollution, inhalation of dust particles, cigarette smoke, and other triggers that can precipitate asthma attacks.^48^ It is well- established that smoking exacerbates inflammation in the airways and worsens asthma conditions. Similarly, the use of electronic cigarettes also acts as a trigger for inflammation and bronchoconstriction,^48^^,^^49^ compromising the innate immune response of the lungs.^25^

A study conducted in 2019 with 1565 adolescents aged between 16 and 19 years in Kuwait aimed to assess the association between the frequency and use of electronic cigarettes with the prevalence of asthma symptoms. The questionnaire applied was developed based on the *National Youth Tobacco Survey* (NYTS) and the *International Study of Asthma and Allergies in Childhood* (ISAAC). The results indicated a significant association between the use of electronic cigarettes and the prevalence of asthma symptoms, irrespective of the use of regular cigarettes or other risk factors. Additionally, there was an association between passive domestic exposure to e-cigarette aerosol and asthma symptoms, highlighting the potential adverse respiratory effects of both primary use and passive exposure to e-cigarettes.^37^

In a systematic review, Rocha and collaborators observed an increased risk of asthma exacerbation in e-cigarette users with chronic asthma, as well as a risk for healthy adolescents.^25^

### *Other damages - explosions and accidental ingestions*

With the increasing use of ENDS, damages other than those related to vapor inhalation have been reported. Particularly notable are injuries from explosions and burns related to batteries, as well as accidental ingestions and intoxications, especially among children who inadvertently ingest nicotine liquid. The National Electronic Injury Surveillance System (NEISS) of the U.S. Consumer Product Safety Commission (CPSC) has provided descriptions and estimated the prevalence of these injuries in subsequent years. The demographic most affected by these incidents was men over 18 years old.^50^, ^51^, ^52^

In the years 2018 and 2019, 65 incidents related to ENDS were documented, including 20 cases of explosion/burn injuries in 2019 and 45 cases of ingestion of e-liquid/use of e-cigarettes by children under 5 years old in 2018–2019. Some of these cases resulted in severe injuries and at least two documented deaths.^50^

The median age of children involved in accidents with nicotine liquid was 1.7 years, with thirty cases (66.6%) occurring in children under 2 years old. Colorful labels depicting fruit and flavored indications are attractive to children. Among the 45 cases of accidental ingestion, three mentioned specific flavors, including cinnamon, raspberry, and grapefruit. Additionally, four cases involved marijuana or THC. The most commonly reported immediate symptom was vomiting, and three children required hospital observation. Furthermore, two infants (aged 11 months to 2 years) were found with devices in their mouths in a smoking position, although ingestion of nicotine liquid was not confirmed.^50^^,^^53^

Some U.S. states have enacted laws requiring child-resistant packaging for nicotine liquid containers, which significantly reduced the average number of exposures from 19.8% to 8.3% in the nine months following the implementation of the law, underscoring the importance of this precaution.^53^

Among the 20 patients treated for explosion and burn injuries, the average age was 38.9 years (ranging from 13 to 66 years), with nine patients requiring hospitalization. In most cases (17 out of 20), the device was in a pocket, and contact with other objects (such as coins) or falling onto the device was predisposed to the accident.^50^^,^^51^

The extremely rapid evolution of these devices' configuration/presentation makes surveillance of these events urgent and even more challenging. These findings support the need for improved regulation and clear, explicit documentation and communication of the imminent risks associated with e-cigarettes.^50^^,^^51^^,^^53^

### *Regulation*

In the USA, the regulatory agency linked to the government's health department, the FDA, established rules for the manufacture, advertising, and sale of electronic cigarettes in 2016, subjecting them to inspection like tobacco products. Since 2019, the US government has raised the minimum age for purchasing tobacco products from 18 to 21, aiming to discourage smoking among teenagers. Despite such measures, NYTS data shows that in 2018, one in five high school students used electronic cigarettes, prompting the American government to declare the use of these devices among young people a national epidemic.^54^

In 2020, the FDA authorized the sale of iQOS® in the American market as a modified-risk tobacco product, creating a perception of reduced harm associated with the product. However, due to the expanding market, data on its effects remains limited, necessitating further research to reach an objective conclusion about the effects of iQOS® on human health and the environment. This is crucial for regulating its use assertively.^14^^,^^15^

In Brazil, the National Health Surveillance Agency (ANVISA) has maintained resolution RDC 46/2009 since 2009. This resolution prohibits the commercialization, import, and advertising of any electronic smoking device, whether containing nicotine or not, throughout the national territory. This regulation has been validated at each review, which occurs periodically.^55^

Recently, on April 19, 2024, ANVISA reviewed its decision and upheld the ban on electronic smoking devices based on current scientific information and feedback from both the scientific and lay communities during public consultation. The updated regulation now prohibits the manufacturing, importation, commercialization, distribution, storage, transportation, and advertising of all electronic smoking devices in Brazil. Consequently, any form of importation, including for personal use and in travelers' carry-on luggage, is prohibited.^55^

It's important to note that while the approved regulation does not extend to the prohibition of individual use, the use of any smoking device in any enclosed public space has been prohibited since 1996, as stipulated by Law 9.294/1996.^55^

Moreover, several Brazilian cities have expanded municipal laws to prohibit smoking in enclosed places, including all electronic smoking devices. An example of this is Curitiba, which enacted Municipal Law 13.254/2009 in May 2024.^56^

### *The role of parents, healthcare professionals, and society*

Medical societies have expressed concerns about the alarmingly increasing use of ENDS and have been conducting lectures and disseminating scientific documents to educate, alert, and inform professionals in all specialties. Documents released by the American Thoracic Society, European Respiratory Society, American Academy of Pediatrics, Pan American Health Organization (PAHO), and the Secretariat of the WHO Framework Convention on Tobacco Control are already available. In Brazil, similar efforts have been made by the Brazilian Society of Pulmonology and Tisiology, Brazilian Society of Pediatrics, National Cancer Institute (INCA), Oswaldo Cruz Foundation (FIOCRUZ), and Brazilian Medical Association (AMB) (refer to the documents of these societies).^57^, ^58^, ^59^, ^60^, ^61^, ^62^, ^63^, ^64^, ^65^, ^66^

Non-governmental organizations, such as Parents Against Vaping E-cigarettes (PAVE) in the United States, have also mobilized to support parents of adolescents addicted to e-cigarettes and to raise awareness among those who may be considering using these devices. PAVE aims to educate parents and communities and advocate for legislation to end the sale of these products.^67^

In the Americas, 21 countries have regulations in place for ENDS, with eight countries completely prohibiting their sale (Argentina, Brazil, Mexico, Nicaragua, Panama, Suriname, Uruguay, and Venezuela). The remaining 13 countries have adopted partial or full regulatory measures, while 14 countries have no regulations for these products.^60^

## Conclusion

Electronic cigarettes, scientifically known as Electronic Nicotine Delivery Systems (ENDS), have emerged as a significant concern for public health authorities in Brazil and other countries where they are freely available. The mounting evidence of their harmful effects in both the short and long term, their appealing design, and the variable mix of nicotine concentrations with other compounds pose a potential risk to the health of the youth.

In Brazil, ANVISA has maintained regulations prohibiting the commercialization, imports, and advertising of ENDS for 15 years, since 2009.

Reports of local damage to the oral cavity, respiratory system, and other organs, as well as the emergence of serious diseases such as EVALI and difficult-to-control asthma, underscore the significant morbidity associated with ENDS use.

Prevention and behavioral counseling emerge as viable interventions to address ENDS use in children and adolescents, as nicotine replacement therapy is not recommended for this population. However, it can be cautiously evaluated to reduce withdrawal symptoms in adolescents.

Being well-informed and capable of providing guidance to colleagues, parents, caregivers, and educators is crucial in protecting the youth from the allure of ENDS. Through education and awareness, the authors can take the first step in safeguarding the health and well-being of future generations from this seductive threat.

## Research funding

No.

## Conflicts of interest

The authors declare no conflicts of interest.

## References

[1] Society Brazilian in Pediatrics. Departments Scientific in Pneumology, Toxicology and Otorhinolaryngology. Electronic nicotine delivery devices (electronic cigarettes and similar): “Wolves in sheep's clothing”. 2018. [Cited 2023 Oct 31] Available at: https://www.sbp.com.br/fileadmin/user_upload/20531e-DocCient_-_DispEletr_entrega_nicotina_e-cigs.pdf.

[2] Brazilian Institute of Geography and Statistics. National Health Survey (2020).

[3] Electronic cigarettes: what do we know? Study on the composition of vapor and damage to health, the role in reducing harm and treating nicotine dependence /National Cancer Institute José Alencar Gomes da Silva; organization Stella Regina Martins. – Rio de Janeiro: INCA; 2016.

[4] Hartmann- Boyce J., McRobbie H., Lindson N., Bullen W., Begh R., Theodoulou A. (2021). Electronic cigarettes for smoke cessation. Cochrane Database Syst Rev.

[5] Soneji S., JL Barrington -Trimis, Wills O.K., Leventhal A.M., Unger J.B., Angeles Gibson Los (2017). Association In between home to use in electronic cigarettes It is Subsequent Smoking among adolescents and young adults: a systematic review and Meta-analysis. JAMA Pediatr.

[6] Banks E., Yazidjoglou A., Brown S., Nguyen M., Martin M., Beckwith K. (2022). E-cigarettes and Health outcomes: Systematic Review of Global evidence. Report to the Australian Department of Health.

[7] Ollila H., Tarasenko Y., Ciobanu A., Lebedeva E., Raitasalo K. (2023). Exclusive and dual use of e-cigarettes among young Europeans in 32 countries with different regulatory scenarios. Tob Control.

[8] Raitasalo K., Bye E.K., Pisinger C., Scheffels J., Tokle R., Kinnunen J.M. (2022). Single, double and triple use of cigarettes, e-cigarettes and Snus among adolescents in the Nordic countries. Int J Environ Res Public Health.

[9] Rowell T.R., Tarran R. Will chronic e-cigarette use cause lung disease? American daily in physiology-lung cell phone it is molecular physiology. 2015;309:L1398–409. 10.1152/ajplung.00272.2015 PMC4683316 26408554

[10] (2023). https://cdn.who.int/media/docs/default-source/tobacco-hq/regulating-tobacco-products/ends-call-to-action-background.pdf.

[11] Bhatnagar A., Whitsel L.P., km Ribisl, Bullen W., Chaloupka F., Piano S.I.R. (2014). Electronic cigarettes. Circulation.

[12] (2019). https://stacks.cdc.gov/view/cdc/103783.

[13] Hendricks K.J., Temples H.S., Wright M.E. (2020). JUULing epidemic among youth: a guide to devices, terminology, and interventions. J Pediatr Health Care.

[14] Başaran R., Güven N.M., Eke BC. (2019). An overview of iQOS ® as a new burn-not tobacco product and its potential effects on human health and the environment. Turk J Pharm Sci.

[15] Yaman B., Akpınar O., Kemal H.S., Cerit L., Yüksek Ü., Söylemez N. (2021). Comparison of IQOS (heated tobacco) and smoking on cardiac functions by two-dimensional speckle tracking echocardiography. Toxicol Appl Pharmacol.

[16] Dutra L.M., Grana R., Glantz S.A. (2017). Philip Morris research on precursors to the modern e-cigarette since 1990. Tob Control.

[17] Boseley S. (2015).

[18] Lik H. Wikipedia. 2015. [Cited 2024 Jun 20]. Available from: https://en.wikipedia.org/wiki/Hon_Lik.

[19] Chun L.F., Moazed F., Calfee C.S., Matthay M.A., Gotts J.E. (2017). Lung toxicity from electronic cigarettes. Am J Physiol Lung Cell Mol Physiol.

[20] Antonini J.M., Taylor M.D., Zimmer A.T., Roberts J.R. (2004). Lung responses to welding fumes: role of metallic constituents. J Toxicol Environ Health A.

[21] Lüdicke F., Picavet P., Baker G., Haziza C., Poux V., Lama N. (2018). Effects of switching to the Menthol Tobacco Heating System 2.2, smoking abstinence, or continued cigarette smoking on clinically relevant risk markers: a randomized, controlled, open-label, multicenter study in sequential confinement and ambulatory settings (Part 2). Nicotine Tob Res.

[22] Ogunwale M.A., Li M., Ramakrishnam Raju M.V., Chen Y., Nantz M.H., Conklin D.J. (2017). Aldehyde detection in electronic cigarette aerosols. ACS Omega.

[23] Znyk M., Jurewicz J., Kaleta D. (2021). Exposure to heated tobacco products and adverse health effects, a systematic review. Int J Environ Res Public Health.

[24] Ruszkiewicz J.A., Zhang Z., Gonçalves F.M., Tizabi Y., Zelikoff J.T., Aschner M. (2020). Neurotoxicity of e-cigarettes. Food Chem Toxicol.

[25] Rocha A.K., Miyawaki A.E., Trombini M.A., Costa Rosa V.A., Prestes R.C., Costa T.B. (2023). Risk of asthma exacerbation in teenage users of electronic nicotine delivery systems: a systematic review and meta-analysis. Arq Asma Allerg Immunol.

[26] Lott E.L., Jones E.B. (2024). StatPearls.

[27] NIDA, 2016. Understanding drug abuse and addiction: what science says. [Cited 2024 Jun 20]. Available from:https://nida.nih.gov/sites/default/files/1921-understanding-drug-abuse-and-addiction-what-science-says.pdf .

[28] Edwards A.C., Kendler K.S. (2011). Nicotine withdrawal-induced negative affect is a function of nicotine dependence and not liability to depression or anxiety. Nicotine Tob Res.

[29] da Silva A.L., Moreira J.C. (2019). Why are electronic cigarettes a threat to public health?. Cad Public Health.

[30] Leventhal A.M., Madden D.R., Peraza N., Schiff S.J., Lebovitz L., Whitted L. (2021). Effect of exposure to e-cigarettes with salt vs free-base nicotine on the appeal and sensory experience of vaping: a randomized clinical trial. JAMA Netw Open.

[31] Yang Q., Ma S., He Y., Qiu Z., Chen J., Shang C (2023). What types of e-liquid products were more likely to offer price promotions?. Tob Control.

[32] Yan D., Wang Z., Laestadius L., Mosalpuria K., Wilson F.A., Yan A. (2023). A systematic review for the impacts of global approaches to regulating electronic nicotine products. J Glob Health.

[33] Rowell T.R., Tarran R. Will chronic e-cigarette use cause lung disease? American daily in physiology-lung cell phone it is molecular physiology. 2015;309:L1398–409. 10.1152/ajplung.00272.2015 PMC4683316 26408554

[34] Siddiqi T.J., Rashid A.M., Siddiqi A.K., Anwer A., Usman M.S., Sakhi H. (2023). Association of electronic cigarette exposure on cardiovascular health: a systematic review and meta-analysis. Curr Probl Cardiol.

[35] Larue F., Tasbih T., Ribeiro P.A., Lavoie K.L., Dolan E., Bacon S.L. (2021). Immediate physiological effects of acute electronic cigarette use in humans: a systematic review and meta-analysis. Respir Med.

[36] Seiler-Ramadas R., Sandner I., Haider S., Grabovac I., Dorner T.E. (2021). Health effects of the use of electronic cigarettes (e- cigarettes) on organic systems and their implications for public health. Wien Klin Wochenschr.

[37] Alnajem A., Redha A., Alroumi D., Alshammasi A., Ali M., Alhussaini M. (2020). Use of electronic cigarettes and passive exposure to their aerosols they are associate with asthma symptoms in between teenagers: the transverse to study. Breathe Res.

[38] Tituana N.Y., Clavijo C.G., Espinoza E.F., Tituana V.A. (2024). Lung injury associated with electronic cigarette use (EVALI). Pneumology.

[39] Sahu R., Shah K., Malviya R., Paliwal D., Sagar S., Singh S. (2023). E-cigarettes and associated health risks: an update on cancer potential. Adv Respir Med.

[40] Winnicka L., Shenoy M.A. (2020). EVALI and the pulmonary toxicity of electronic cigarettes: a review. J Gen Intern Med.

[41] Kligerman S., Raptis C., Larsen B., Henry T.S., Caporale A., Tazelaar H. (2020). Radiological, pathological, clinical and physiological outcomes of electronic cigarette or lung injury associated with vaping product use (EVALI): evolution of knowledge and remaining questions. Radiology.

[42] Layden J.E., Ghinai I., Pray I., Kimball A., Layer M., Tenforde M.W. (2020). Pulmonary illness related to e-cigarette use in Illinois and Wisconsin - final report. N Engl J Med.

[43] Henry T.S., Kanne J.P., Kligerman S.J. (2019). Imaging of vaping-associated lung disease. N Engl J Med.

[44] Butt Y.M., Smith M.L., Tazelaar H.D., Vaszar L.T., Swanson K.L., Cecchini M.J. (2019). Pathology of vaping-associated lung injury. N Engl J Med.

[45] Kaur G., Lungarella G., Rahman I. (2020). SARS-CoV-2 COVID-19 susceptibility and lung inflammatory storm by smoking and vaping. J Inflamm.

[46] Li X., Geng M., Peng Y., Meng L., Lu S. (2020). Molecular immune pathogenesis and diagnosis of COVID-19. J Pharm Anal.

[47] Blount B.C., Karwowski M.P., Morel-Espinosa M., Rees J., Sosnoff C., Cowan E. (2019). Evaluation of bronchoalveolar lavage fluid from patients in an outbreak of e-cigarette, or vaping, product use-associated lung injury - 10 states, August-October 2019. MMWR Morb Mortal Wkly Rep.

[48] Schweitzer R.J., Wills T.A., Tam E., Pagano I., Choi K (2017). E-cigarette use and asthma in a multiethnic sample self teenagers. Previous Med.

[49] Malta D.C., Gomes C.S., Alves F.T., Oliveira P.P., Freitas P.C., Andreazzi M. (2022). The use of cigarettes, hookah, electronic cigarettes, it is other tobacco indicators in between Brazilian schoolchildren: data from the 2019 National School Health Survey. Rev Bras Epidemiol.

[50] Rossheim M.E., McDonald K.K., Soule E.K., Gimm G.W., Livingston M.D., Barnett T.E. (2020). Electronic cigarette explosion/burn and poisoning related emergency department visits, 2018-2019. Am J Emerg Med.

[51] Wiener R.C., Lundstrom E.W (2024). Injuries from electronic cigarettes, and cigarette/cigar-related paraphernalia, NEISS, 2012-2022. PLoS One.

[52] McKenna Jr L.A. U.S. Fire Administration report. Electronic cigarette fires and explosions in the United States 2009–2016. [Cited 2024 Jun 20]. Available from: https://www.usfa.fema.gov/downloads/pdf/publications/electronic_cigarettes.pdf.

[53] Govindarajan P., Spiller H.A., Casavant M.J., Chounthirath T., Smith G.A. (2018). E-Cigarette and liquid nicotine exposures among young children. Pediatrics.

[54] Cullen K.A., Ambrose B.K., Gentzke A.S., Apelberg B.J., Jamal A., King B.A. (2018). Field notes: use of electronic cigarettes and any tobacco product among elementary and secondary school students - United States, 2011-2018. MMWR Morb Mortal Wkly Rep.

[55] Brazil. National Health Surveillance Agency. Resolution of the Collegiate Board - RDC no 46, of August 28, 2009. Prohibits the commercialization, import and advertising in any devices electronics for smoke, acquaintances as cigarette electronic. [Internet]. Daily Official from the Unity 2009. [Cited 2024 Mar 17]. Available from:http://portal.anvisa.gov.br/documents/10181/2718376/RDC_46_2009_COMP.pdf/214 8a322-03ad-42c3-b5ba-718243bd1919.

[56] Câmara Municipal de Curitiba. Saúde quer mais informações sobre ampliação da Lei Antifumo. [Cited 2024 Jun 20]. Available from:https://www.curitiba.pr.leg.br/informacao/noticias/saude-quer-mais-informacoes-sobre-ampliacao-da-lei-antifumo.

[57] American Thoracic Society, ATS. [Cited 2024 Jun 20]. Available from: https://site.thoracic.org.

[58] European Respiratory Disease, ERS. [Cited 2024 Jun 20]. Available from: https://www.ersnet.org.

[59] American Academy of Pediatrics, AAP. [Cited 2024 Jun 20]. Available from: https://www.aap.org.

[60] Organização Pan-Americana de Saúde, OPAS. [Cited 2024 Jun 20]. Available from: https://www.paho.org/pt/brasil.

[61] World Health Organization Framework Convention on Tobacco Control, WHO FCTC. [Cited 2024 Jun 20]. Available from: https://fctc.who.int/who-fctc/overview.

[62] Sociedade Brasileira de Pneumologia e Tisiologia, SBPT. [Cited 2024 Jun 20]. Available from: https://sbpt.org.br/portal/.

[63] Sociedade Brasileria de Pediatria, SBP. [Cited 2024 Jun 20]. Available from: https://www.sbp.com.br.

[64] Instituto Nacional de Cancer, INCA. [Cited 2024 Jun 20]. Available from: https://www.gov.br/inca/pt-br.

[65] Fundação Osvaldo Cruz, FIOCRUZ. [Cited 2024 Jun 20]. Available from: https://portal.fiocruz.br.

[66] Associação Médica Brasileira, AMB. [Cited 2024 Jun 20]. Available from: https://amb.org.br.

[67] Parents Against Vaping E-Cigarettes, PAVE. [Cited 2024 Jun 20]. Available from: https://www.parentsagainstvaping.org.

